# Thrombo-inflammation linking androgen suppression with cardiovascular risk in patients with prostate cancer

**DOI:** 10.1186/s40959-024-00278-2

**Published:** 2024-12-05

**Authors:** Antonia Beitzen-Heineke, David R. Wise, Jeffrey S. Berger

**Affiliations:** 1 Department of Medicine, New York University Grossman School of Medicine, 530 First Avenue, Skirball 9R, New York, NY 10016 USA; 2 Department of Oncology and Hematology, University Medical Center Hamburg-Eppendorf, Hamburg, Germany; 3 Department of Medicine, Laura & Isaac Perlmutter Cancer Center, NYU Langone Health, New York, NY USA

**Keywords:** Androgen deprivation therapy, Prostate cancer, Cardiooncology, Cardiotoxicity, Cardiovascular toxicity, Atherosclerosis, Platelet activity, Platelet aggregation, Inflammation, Thromboinflammation

## Abstract

Androgen deprivation therapy (ADT), a key element of prostate cancer treatment, is associated with increased risk for cardiovascular morbidity and mortality. The underlying mechanisms include adverse metabolic alterations, but further mechanisms are likely. Animal studies suggest increased progression of atherosclerosis in androgen deprived conditions. Based on in vitro studies, lack of androgens may modulate immune cells including monocytes, macrophages, and T-cells towards a pro-inflammatory phenotype and pro-atherogenic function. As a novel aspect, this review summarizes existing data on the effect of androgens and androgen deprivation on platelet activity, which play a major role in inflammation and in the initiation and progression of atherosclerotic lesions. Testosterone modulates platelet aggregation responses which are affected by dose level, source of androgen, and age. Data on the effects of ADT on platelet activity and aggregation are limited and conflicting, as both increased and decreased aggregation responses during ADT have been reported. Gaps in knowledge about the mechanisms leading to increased cardiovascular risk during ADT remain and further research is warranted. Improved understanding of pathogenic pathways linking ADT to cardiovascular risk may help identify clinically useful diagnostic and prognostic biomarkers, and accelerate finding novel therapeutic targets, and thus optimize prostate cancer treatment outcomes.

## Background

Prostate cancer is the most common non-cutaneous cancer in men. As prostate cancer growth is hormone-dependent, androgen deprivation therapy (ADT) is the key element of prostate cancer treatment in locally advanced and metastatic disease. In the prior decades, as prostate cancer treatment efficacy increased and survival improved, an association of ADT and cardiovascular disease (CVD) became evident [1]. Various observational studies reported an association of ADT with atherosclerotic plaque progression and instability, coronary artery disease, and stroke [1]. Adverse metabolic changes associated with ADT contribute to increased CVD risk but additional pathogenic mechanisms of cardiovascular toxicity are likely [2]. Evidence suggests that androgens and ADT have effects on local inflammatory processes that contribute to atherosclerotic plaque development and instability [3]. In addition, the majority of prostate cancer patients present with pre-existing cardiovascular risk factors at diagnosis and the relative CVD risk is increased among all men with prostate cancer, including those undergoing active surveillance [4, 5]. Hence, CVD is the leading cause of death in prostate cancer patients [6]. To lower the risk of CVD during ADT, optimization of cardiovascular risk factor control is recommended [7, 8]. Moreover, a precise understanding of the mechanisms of the increased CVD risk is essential to guide prevention strategies and optimize prostate cancer treatment outcome. This review highlights inflammatory mechanisms involved in ADT-associated CVD and elaborates on the effects of ADT on platelets and thrombo-inflammation which play a major role in atherogenesis and CVD.

## Prostate cancer – epidemiology

The incidence of prostate cancer varies depending on geographical areas, age of the population and ethnicity with highest incidences found in Australia/New Zealand, Northern Europe, North America and the Caribbean [9–11]. In the US, around 300,000 new cases of prostate cancer will be diagnosed in 2024. In the past decade, the incidence rate has increased 3% per year [12]. After a drastic decline in prostate cancer mortality mainly due to effective screening measures leading to earlier detection, mortality rates have stabilized since 2017. The majority of prostate cancer patients (83%) are diagnosed in local or regional disease stages with excellent survival rates of nearly 100% at 5 years [12]. Among these patients, ADT has proven to improve survival in men with high-risk localized and locally advanced disease treated with radiotherapy. In advanced disease stages, ADT is the cornerstone of treatment for all patients [13]. 

### Types of ADT

**Fig. 1:**
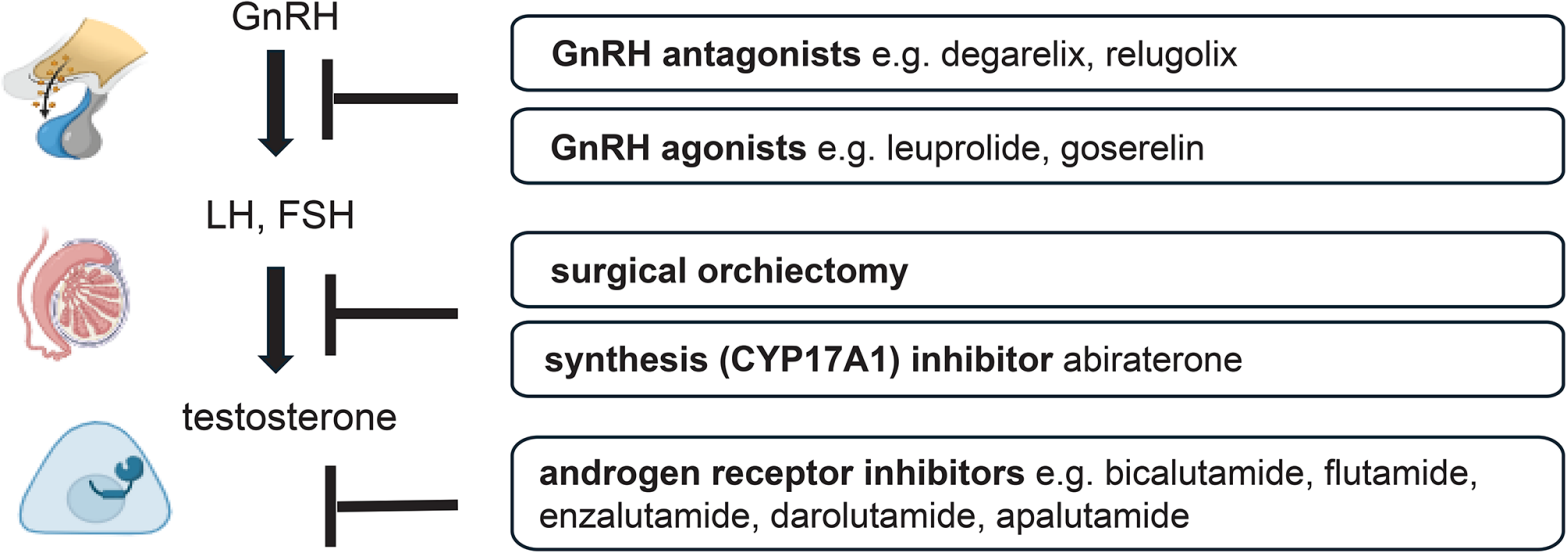
Visual summary of the different types of androgen pathway directed therapies for prostate cancer treatment. Classical androgen deprivation therapy comprises Gonadotropin releasing hormone (GnRH) agonists and GnRH antagonists. Further options include surgical orchiectomy, CYP17A inhibition, and androgen receptor inhibition. Figure created with BioRender.com

Androgen deprivation can be achieved by surgical or pharmacological castration (Fig. 1). Most commonly, suppression of testosterone secretion is attained using gonadotropin-releasing hormone (GnRH) agonists or GnRH antagonists which is traditionally referred to as ADT. In addition, androgen signaling can be inhibited at the receptor level using antiandrogens. GnRH agonists cause an initial increase of luteinizing hormone (LH) and follicle-stimulating hormone (FSH) secretion causing a temporary flare before chronic exposure to GnRH agonists results in suppression of LH and FSH secretion and consequently a suppression in testosterone production. To mitigate the clinical flare-up, GnRH agonists are usually combined with an antiandrogen (e.g. bicalutamide) for the first weeks of treatment. GnRH antagonists suppress testosterone production without causing an initial flare. In contrast to GnRH agonists which are available in 3-month and 6-month depot-formulations, GnRH antagonists injections need to be applied monthly. However, recently the first oral formulation of a GnRH antagonist, relugolix, was approved by the FDA and EMA, and is taken on a daily basis [9]. 

In the past decade, several new hormonal agents (NHA) were added to the treatment landscape: the androgen synthesis inhibitor abiraterone (CYP17A1 inhibitor) and the next-generation androgen receptor inhibitors enzalutamide, apalutamide and darolutamide. These NHA are usually administered in addition to ADT in castration resistance or metastatic disease. The implementation of NHA has led to prolonged survival outcomes of many years even in advanced stages [14, 15]. Therefore, treatment related side effects and competing health risks are of particular importance in prostate cancer patients treated in curative intent as well as in advanced disease stages [16]. This review focusses on the mechanisms of the increased CV risk of traditional ADT.

### ADT and CV risk

Numerous observational studies and meta-analyses of observational studies reported increased CV morbidity including coronary heart disease, myocardial infarction, and stroke, as well as increased CV mortality in association with ADT [2, 5, 17–20]. In meta-analyses of randomized controlled trials, no significant increase in CV mortality was found in men undergoing ADT [21–23]. The strongest evidence of increased CV morbidity and mortality is derived from observational studies of GnRH agonists. Data from randomized controlled studies comparing GnRH agonists versus antagonists suggest that GnRH antagonists are associated with fewer CV events compared to GnRH agonists [24, 25]. With the aim to provide evidence for the relative cardiovascular safety of GnRH agonists and antagonists, the PRONOUNCE study prospectively compared major adverse cardiovascular events (MACE) in prostate cancer patients with preexisting atherosclerotic CVD randomized to receive GnRH agonists versus GnRH antagonists. The study was ended prematurely due to the slower than anticipated patient accrual and the low number of events that occurred, and no difference in MACE (composite of death, myocardial infarction, or stroke) between groups was observed. Of note, in this study, all patients were seen by a cardiologist prior to treatment initiation and prescription of lipid lowering medication (84%), agents acting on the renin-angiotensin system (73%), and beta-blockers (69%) was high [26]. With regards to timing and risk for CVD during ADT, a Swedish cohort study described the highest risk for cardiovascular events for patients with preexisting CVD was within the first six months of androgen deprivation including treatment with GnRH agonists, androgen receptor antagonists or surgical orchiectomy [27]. However, data on CV risk during long-term treatment with ADT are limited. A meta-analysis of eight observational studies found that GnRH agonist treatment increases the risk of any type of non-fatal CVD by 38% compared to patients not treated with ADT and associations with non-fatal and fatal myocardial infarction were even stronger at 57% and 51%, respectively [28]. Identifying the subset of men at highest risk of an adverse CV risk remains challenging although patients with prior CV events seem to be at highest risk [27]. Whether mechanisms leading to early versus late CV events on ADT differ remains unknown as well. However, the initial surge of LH, FSH and consequently testosterone during GnRH agonist treatment may contribute to the risk of CVD during early phases of ADT whereas other mechanisms may be more relevant in the long-term.

### Mechanisms of increased CV risk

**Fig. 2:**
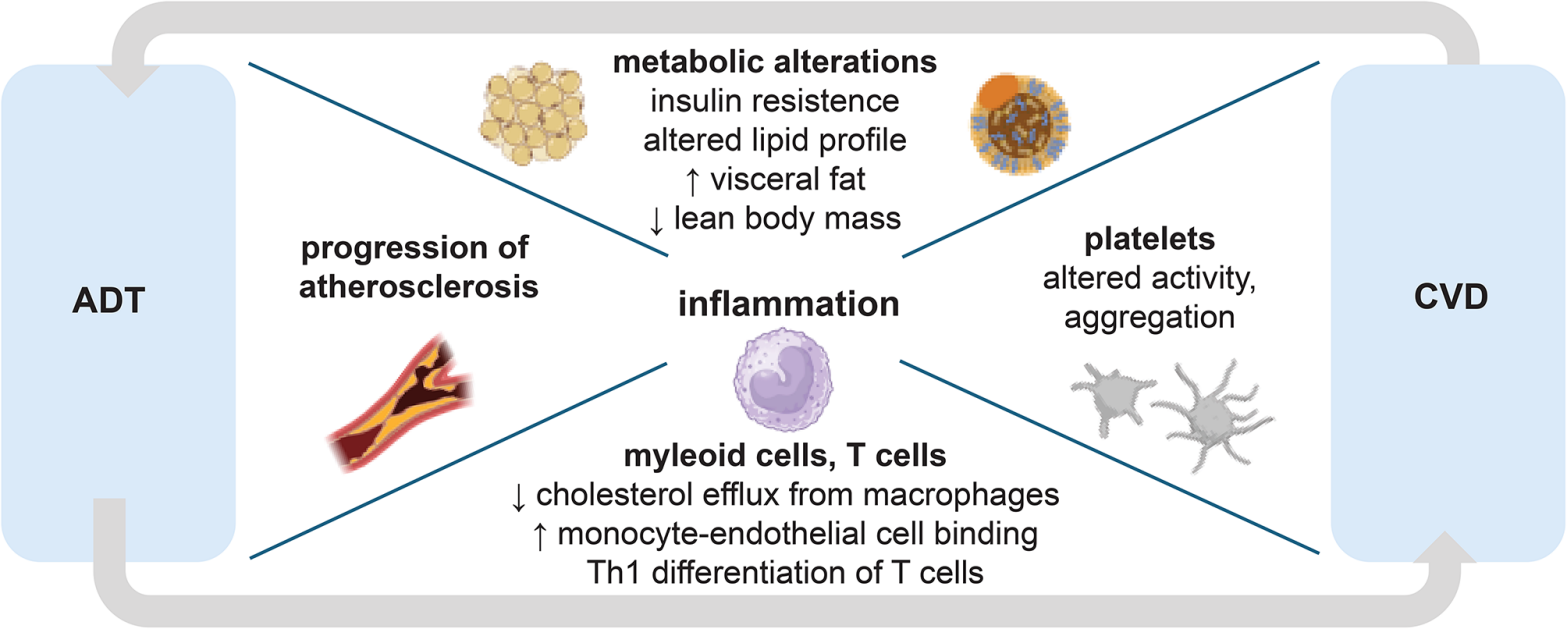
Schematic overview of potential mechanisms of increased risk for cardiovascular disease (CVD) during androgen deprivation therapy (ADT). ADT associated metabolic alterations contribute to increased CVD risk. Moreover, ADT may lead to pro-inflammatory changes in macrophages, monocytes and T-cells which further contribute to progression of atherosclerosis. Platelet activity and aggregation is modulated by androgens and may also play a role in ADT-associated CV risk. Figure created with BioRender.com

Underlying mechanisms likely include alterations in hormones and cytokines and direct effects on immune cells that ultimately result in increased inflammation. While precise mechanisms of the increased CV risk remain incompletely understood, different pathways have emerged to play a role (Fig. 2). A multitude of adverse metabolic alterations are associated with ADT which have been discussed in previously published reviews [2, 29]. The following sections concentrate on studies suggesting pro-atherogenic effects of ADT and underlying inflammatory pathways. As a novel aspect, we review thrombo-inflammation and the potential mechanistic role of platelets influencing the relationship between ADT and CV risk.

### ADT and atherosclerosis

Murine studies suggest that androgen deprivation worsens atherosclerosis, inducing larger atherosclerotic lesions in orchiectomized mice and in androgen receptor knockout mice compared to sham-treated and wild-type mice, respectively [37, 38]. Testosterone supplementation reduced atherosclerosis in both androgen-receptor knockout mice on apolipoprotein E-deficient background and wild-type mice but the extent of atheroprotection of testosterone was lower in androgen-receptor knockout mice [37]. In orchiectomized mice, pro-atherogenic effects were reversed when testosterone or estradiol was administered, but not when testosterone was supplemented concomitant with anastrozole, a selective aromatase inhibitor that prevents conversion of testosterone to estrogens [38]. Another in vivo study showed increased atherosclerosis after ubiquitous knockout of the androgen receptor which was accompanied by a pronounced drop in testosterone levels but showed reduced atherosclerosis in atherogenic mice selectively lacking the androgen receptor in monocytes/macrophages concomitant with unchanged testosterone levels [39]. Altogether, these studies suggest that testosterone has atheroprotective effects via androgen receptor dependent and androgen receptor independent mechanisms including aromatization of testosterone to estradiol. Moreover, a recent study demonstrated that long- and short-term FSH elevations resulting from orchiectomy and GnRH agonist treatment, respectively, contribute to atherosclerosis progression [40]. These findings could explain the lower cardiovascular risk of GnRH antagonist treatment, which does not cause FSH elevations.

### ADT and macrophages

In vitro, incubation of human monocyte-derived macrophages in the presence of testosterone at increasing concentrations increased cholesterol efflux, thus facilitating removal of excess cholesterol from atherosclerotic lesions [41]. In another study, pro-inflammatory cytokines were decreased when monocyte-derived macrophages were cultured in the presence of testosterone and expression of vascular adhesion molecule-1 (VCAM-1) by endothelial cells, which is important for monocyte adhesion, was decreased [3]. These studies suggest that low testosterone levels may promote inflammation and monocyte-endothelial cellbinding. More recently, FSH was described to exacerbate endothelial inflammation in the presence of tumor necrosis factor α as demonstrated by increased expression of VCAM-1, E-selectin, and monocyte chemoattractant protein-1 (MCP-1) in endothelial cells. Moreover, monocyte-endothelial cell binding was enhanced after FSH treatment and pro-inflammatory effects of FSH on macrophages were observed [40]. In contrast to the in vitro data, in prospective clinical studies a 12 month treatment with a GnRH agonist and a 12 week treatment with a GnRH agonist concomitant with bicalutamide, respectively, showed no increased levels of the inflammatory markers C-reactive protein and resistin in peripheral blood of prostate cancer patients [36, 42]. 

### ADT and T cells

Also, T cells express the GnRH receptor and GnRH receptor activation on T cells was described to result in expansion and differentiation into pro-inflammatory Th1 T cells in rats [43, 44]. Th1 T cells in turn are important macrophage activators. Thereby, GnRH agonist binding to T cells in atherosclerotic plaques may promote atherosclerotic plaque destabilization [45]. Cardiovascular events peak during the first 6 months of GnRH agonist treatment supporting the hypothesis that mechanisms other than rather long-term metabolic changes contribute to the increased CV risk during GnRH agonist treatment [27]. Furthermore, T cells express the androgen receptor and androgens exert inhibitory effects on Th1 differentiation [3, 46]. While pro-inflammatory Th1 cells promote progression of atherosclerosis, Th2 and Th17 cells have been associated with both proatherogenic and atheroprotective properties. T regulatory cells (Tregs) are considered atheroprotective and an inverse relationship of Treg numbers was reported in patients with unstable coronary artery disease [47–49]. The impact of alterations in T cell numbers and T cell differentiation on increased CV risk during ADT remains to be investigated.

### Platelets and thrombosis

In addition to their key role in thrombosis and hemostasis, platelets play a major role in inflammation and in the initiation and progression of atherosclerotic lesions, and increased platelet activity is associated with acute and chronic CVD [50–54]. Consistent with other atherosclerosis-associated cell types, platelets express the androgen receptor, and androgen levels influence platelet activity [3]. 

Investigations on the effect of testosterone and its derivatives on platelet phenotype independent of prostate cancer show conflicting results. In healthy men aged 60–65 years, testosterone and dihydrotestosterone plasma concentrations were inversely associated with platelet activity and reactivity [55]. Furthermore, in vitro treatment of platelets with testosterone and dihydrotestosterone inhibited aggregation in response to the agonists arachidonate and collagen, respectively [55]. In contrast, in a small placebo-controlled study of healthy men aged 21–35 years, testosterone treatment increased thromboxane A_2_ (TXA_2_) receptor density on platelets and aggregation responses to different concentrations of a TXA_2_ mimetic [56]. In a longitudinal follow-up study of 15 men with Klinefelter syndrome presenting with hypogonadotropic hypogonadism, maximum platelet aggregation responses were not altered after 18 months of testosterone supplementation compared to baseline. Compared to a male reference group, aggregation responses to thrombin receptor activation protein-6 (TRAP-6) and arachidonic acid were significantly lower in men with Klinefelter syndrome at baseline but no differences between groups were found at follow-up after implementation of testosterone supplementation [57]. In contrast, another cross-sectional study that used a variety of agonists compared 23 patients with Klinefelter syndrome under testosterone replacement therapy to 46 age-matched healthy males and found increased platelet reactivity in men with Klinefelter syndrome on testosterone supplementation, as demonstrated by a reduced minimal agonist concentration needed to achieve an adequate platelet aggregation [58]. In addition, various animal studies have provided evidence that supraphysiological levels of testosterone may enhance platelet activity [59]. In vitro, androgen receptor expression in CD34^+^ stem cell derived megakaryocytes was shown to be regulated by testosterone. Treatment with low concentrations of testosterone (1 to 10 nmol/L) resulted in upregulation of androgen receptor expression whereas high concentrations of testosterone (100 nmol/L) caused a downregulation of the androgen receptor [60]. Another in vitro study demonstrated that expression of tissue factor pathway inhibitor (TFPI) and tissue plasminogen activator (tPA) was increased in endothelial cells under physiological levels of testosterone, thereby enhancing anticoagulant activity, whereas supraphysiological concentrations of testosterone had contrasting effects [61]. Altogether, the relationship of testosterone levels on platelet activity and thrombosis seems to be non-linear and may depend on age and source of androgen (exogenous vs. endogenous).

Data on platelet function and activity in androgen deprived conditions are scarce and contradictory. In a small case-control study, platelet TXA_2_ receptor density and aggregation responses to a TXA_2_-mimetic and thrombin were studied in prostate cancer patients after surgical orchiectomy or on ADT (*n* = 8) compared to a non-castrated control group (*n* = 7). TXA_2_ receptor density and the maximum aggregation responses to both agonists were significantly reduced in castrated men. But, no changes in TXA_2_ receptor affinity and EC50 aggregation responses were observed between groups [62]. In contrast, platelet aggregation was enhanced in castrated rats compared to sham-operated mice and dihydrotestosterone replacement to physiological levels in castrated animals suppressed platelet aggregation again [63, 64]. In vitro, androgen receptor negative and -inhibited prostate cancer cell lines were shown to increase prothrombin expression and thereby induce platelet aggregation and hypercoagulability as opposed to androgen receptor-positive prostate cancer cell lines [65]. 

Interestingly, in men with castration resistant prostate cancer on ADT, platelets were described to synthesize testosterone from its precursor cholesterol, thereby contributing to androgen receptor signaling in castration resistant disease [66]. Moreover, antiplatelet and anticoagulant therapy was associated with improved progression free survival in prostate cancer patients receiving primary radiotherapy for prostate cancer in curative intent [67]. These data hint towards a role of platelets on prostate cancer outcome. In summary, androgen pathway signaling has effects on platelet activity which are incompletely understood, and further studies are warranted to decipher the interconnection of platelets, ADT and CVD risk.

Our group and others have demonstrated that leukocyte-platelet aggregates (LPA) are robust biomarker of platelet activity, represent the crossroad between thrombosis and inflammation, and are increased in patients with cardiovascular disease [68–70]. LPA formation is mediated by P-selectin expression on activated platelets which binds to P-selectin glycoprotein ligand-1 (PSGL-1) on monocytes and neutrophils [71]. Interestingly, LPA in women with CVD were higher than in men with CVD and LPA in healthy women varied during the menstrual cycle, suggesting a direct effect of sex hormones on LPA formation [72, 73]. Whether LPA formation is increased and contributes to increased CV risk in prostate cancer patients undergoing ADT remains to be elucidated.

### Ongoing studies

Currently, the open-label randomized REPLACE-RV study aims to prospectively evaluate the risk for major adverse cardiovascular events in patients with prostate cancer treated with the GnRH antagonist relugolix compared to the GnRH agonist leuprolide acetate. Concomitantly, detailed information on clinical data and cardiovascular risk will be collected. This study may help to further understand the CV risk of different ADT regimens and thereby may help to guide prostate cancer treatment in patients at increased risk for CVD. Also currently recruiting, the Add-Aspirin study (NCT02804815) is a phase III placebo-controlled randomized trial investigating whether Aspirin use after standard treatment prevents recurrence and prolongs survival in non-metastatic cancers including prostate cancer.

## Conclusions

A myriad of interconnections exist between cardiovascular risk factors, CVD, androgen deprivation therapy and prostate cancer. Gaps in knowledge of the pathomechanisms of increased CVD risk during ADT remain and identification of novel pathways and biomarkers could help guide prophylactic strategies in this patient group. Depicting the role of platelets in CVD risk and prostate cancer progression during ADT could potentially lay the basis for targeted therapies.

## Data Availability

No datasets were generated or analysed during the current study.

## Abbreviations

ADT: Androgen deprivation therapy
CVD: Cardiovascular disease
EC50: Half maximal effective concentration
FSH: Follicle-stimulating hormone
GnRH: Gonadotropin-releasing hormone
LH: Luteinizing hormone
LPA: Leukocyte-platelet aggregates
MACE: Major adverse cardiovascular events
MCP-1: Monocyte chemoattractant protein-1
NHA: New hormonal agent
PSGL-1: P-selectin glycoprotein ligand-1
TFPI: Tissue factor pathway inhibitor
Th: T helper
tPA: Tissue plasminogen activator
TRAP 6: Thrombin receptor activation protein-6
Treg: T regulatory cells
TXA_2_: Thromboxane A_2_
VCAM-1: Vascular adhesion molecule-1
